# *FGFR2/3 g*enomic alterations and response to Enfortumab Vedotin in metastatic urothelial carcinoma

**DOI:** 10.1002/bco2.125

**Published:** 2021-11-10

**Authors:** Elio Adib, Talal El‐Zarif, Rohit K. Jain, William P. Skelton, Dory Freeman, Catherine Curran, Elie W. Akl, Amin H. Nassar, Praful Ravi, Charlene Mantia, David J. Kwiatkowski, Toni K. Choueiri, Guru P. Sonpavde

**Affiliations:** ^1^ Department of Medicine, Brigham and Women's Hospital Harvard Medical School Boston Massachusetts USA; ^2^ Lank Center for Genitourinary Oncology Dana‐Farber Cancer Institute Boston Massachusetts USA; ^3^ H. Lee Moffitt Cancer Center and Research Institute Tampa Florida USA

**Keywords:** biomarkers, bladder cancer, enfortumab vedotin, genomics, targeted therapy

## 1 INTRODUCTION

The therapeutic landscape of metastatic urothelial carcinoma (mUC) has dynamically changed with the recent approval of multiple new agents. Enfortumab Vedotin (EV), an anti‐Nectin‐4 antibody‐drug conjugate (ADC) was approved for patients progressing after platinum‐based chemotherapy and PD1/L1 inhibitors regardless of biomarker status. Moreover, Erdafitinib, an FGFR inhibitor (FGFRi) is the first targeted agent approved for mUC with genomic alterations (GAs) like somatic activating mutations or fusions in *FGFR2/3* progressing post‐platinum therapy.^1^, ^2^

Following the advent of switch maintenance avelumab in those with stable or responding disease after platinum‐based chemotherapy, optimal selection of patients for third‐line therapy with EV or erdafitinib is unclear.^3^ There are no data supporting any biomarker with response to EV, which poses challenges in the choice of therapy. To provide more data that can inform management of mUC in this setting, we investigated the activity of EV based on the presence or absence of activating somatic *FGFR2/*3 GAs.

## 2 METHODS

We conducted a retrospective study to assess the objective response rate (ORR), overall survival (OS), and progression‐free survival (PFS) in all mUC patients who had received EV at Dana‐Farber Cancer Institute (DFCI) and Moffitt Cancer Center (MCC) between 2017 and 2021 and had available targeted panel next‐generation sequencing data from tissue samples using Oncopanel^4^ at DFCI and FoundationOne^5^ at MCC. *FGFR2/3* GAs included activating gene fusions and hotspot mutations which were confirmed to be “pathogenic” or “likely pathogenic” by the Sorting Intolerant from Tolerant (SIFT)^6^ algorithm and have a gain‐of‐function annotation by OncoKB.^7^

ORR was determined by physicians according to response evaluation criteria in solid tumors (RECIST v1.1). OS was defined as survival from the time of EV initiation to the time of death or censored at date of last follow‐up. PFS was defined as duration from the time of EV initiation to the time of progression or death, whichever came first. Multivariable cox regression analysis was used to examine associations between *FGFR2/3* alterations and clinical outcomes. Prior therapy, non‐urothelial histology component, metastasis pattern (liver, non‐liver visceral, and soft‐tissue/lymph node only), ECOG‐performance status (PS), hemoglobin and neutrophil‐to‐lymphocyte ratio at time of EV initiation were used as covariates. ORR analysis was performed using a logistic regression model adjusting for the aforementioned covariates. All *p‐*values were two‐tailed; significance was set at *α* = 0.05.

## 3 RESULTS

Sixty patients were included in the analysis: 47 from DFCI and 13 from MCC. The median age was 70.5 (48–88) years and 44 (73%) were male (Table 1, supporting information Tables S1 and S2). Forty‐seven patients had received both platinum‐based chemotherapy and PD1/L1 inhibitors with a median of 2 (0–3) lines of systemic therapy prior to EV. Most patients had an ECOG‐PS score of 0–1 (53/60, 88.3%), and 26 (43.3%) had liver metastasis (Table 1 and supporting information Table S1). Overall, 13 patients had *FGFR2/3* GAs. Nine patients had confirmed activating hotspot *FGFR3* mutations (p.R248C, p.S249C, p.G370C, or p.Y373C), and one had confirmed activating hotspot *FGFR2* mutation (p.N549K, supporting information Table S1). Three patients had *FGFR3‐TACC3* fusions (supporting information Table S1).

**TABLE 1:** Baseline clinical and demographic characteristics of 60 mUC patients treated with EV

	*N* = 60	
	*N* (median)	% (range)
Treatment site		
DFCI	47	‐
MCC	13	‐
Age at EV start	70.5	48–88
Sex		
Male	44	73.3
Female	16	26.7
Primary site		
Bladder	46	76.7
Upper tract	13	21.7
Urachus	1	1.6
Histology		
Pure urothelial	44	73.3
Mixed predominant urothelial	16	26.7
ECOG‐PS		
0	31	51.7
1	22	36.7
≥2	7	11.6
Metastatic site		
Lymph node +/− soft‐tissue	13	21.7
Nonliver visceral +/− other	31	51.7
Liver +/− other	16	26.6
Neutrophil‐to‐lymphocyte ratio	5.6	0.8–46.7
Hemoglobin level	11.7	7.7–16
Prior chemotherapy		
Yes	49	81.7
No	11	18.3
Prior ICI treatment		
Yes	57	95.0
No	3	5.0
*FGFR2/3* GA		
No	47	78.3
Yes	13	21.7

Abbreviations: DFCI, Dana‐Farber Cancer Institute; EV, Enfortumab Vedotin; GA, genomic alteration; MCC, Moffitt Cancer Center; mUC, metastatic urothelial carcinoma.

Of 54 patients evaluable for ORR, 6 had complete response (CR), 24 had partial response (PR), 21 had stable disease (SD), and 3 had progressive disease (PD) as the best response. Patients with *FGFR2/3* activating mutations exhibited an ORR that was not statistically different compared to patients without mutations: 5/11 (45%; 95% CI: 17–77%) versus 25/43 (58%; 95% CI: 42%–73%), respectively (multivariable logistic regression *p‐*value = 0.43). All five *FGFR* *2/3*‐altered patients who responded had hotspot mutations, and none of the three patients with *FGFR3‐TACC3* fusion responded. Eight of 13 patients with *FGFR2/3* GAs received an FGFRi: one prior to EV who responded, while 7 were treated with FGFRi post‐EV and none responded (0/7).

**FIGURE 1:**
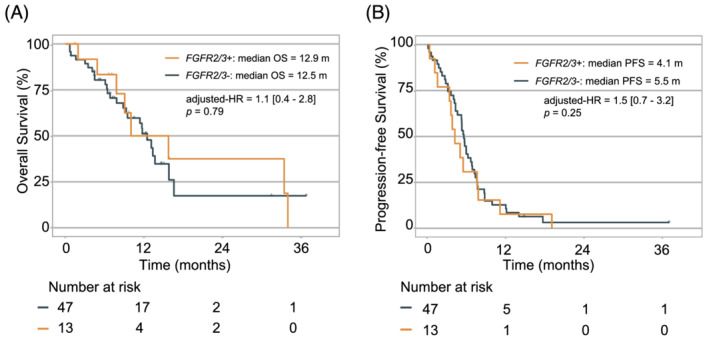
Overall (A) and progression‐free (B) survival post‐Enfortumab Vedotin (EV) treatment* in *FGFR2/3+* and *FGFR2/3−* tumors. *All patients received single agent EV 1.25 mg/kg IV over 30 min on days 1, 8, and 15 of every 28‐day cycle

After adjusting for covariates, mUC patients with and without *FGFR2/3* GAs had similar OS (adjusted HR = 1.1 [95%CI: 0.4–2.8]; *p =* 0.79; median OS _*FGFR2/3*+_ = 12.9 [9.1 – NR] months versus median OS _*FGFR2/3*‐_ = 12.5 [9.5 – NR] months; Figure 1A; supporting information Table S3). Similarly, PFS was not significantly different between the two groups (adjusted HR = 1.5 [95%CI: 0.7–3.2]; *p =* 0.25; median PFS _*FGFR2/3*+_ = 4.1 [3.3 – NR] months versus median PFS _*FGFR2/3*‐_ = 5.5 [4.5–6.8] months; Figure 1B).

## 4 DISCUSSION

*FGFR* activating alterations occur in 15%–20% of mUCs.^8^ Based on our findings, activating GAs in *FGFR2/3* did not significantly influence clinical outcomes, that is, ORR, PFS, and OS post‐EV. We further showed that none of the seven patients responded to an FGFRi administered post‐EV, whereas the only patient who received an FGFRi prior to EV responsed.^2^ Our findings suggest that prior EV treatment may desensitize mUC against subsequent FGFRi, but the underlying biology is not well understood. Interestingly, our data suggest that those with *FGFR2/3* activating fusions may be less sensitive to EV (0/3 responders) than those with FGFR2/3 mutations (5/8 responders), similar to the trend observed with erdafitinib.^2^ These data and the optimal sequencing of EV and erdafitinib warrant assessment in a larger cohort. Interestingly, among TCGA samples of muscle invasive bladder cancer, expression of *NECTIN4* was twofold higher in patients with *FGFR2/3* driver GAs (log‐fold change = 1.08, *q =* 2.5e‐8), suggesting a possible crosstalk between the receptors.^8^ Indeed, the combination of EV and FGFRi may warrant investigation.

Our work has multiple limitations: First, it is a retrospective analysis of clinical outcomes in a modest number of patients. Second, patients treated at two tertiary academic centers are likely enriched for unusual clinical features and more aggressive disease. Third, DNA‐based sequencing panels are suboptimal at detecting gene fusions such as *FGFR3‐TACC3* and RNA‐based methods may be more useful. However, only known bonafide pathogenic mutations and fusions were identified in the GA + group in our study. Finally, some tumors were sequenced after systemic treatment (immune‐checkpoint inhibitors or chemotherapy).

In conclusion, EV could be an acceptable option for patients with *FGFR2/3* GAs directly after progressing on platinum‐based chemotherapy and PD1/L1 inhibitor immunotherapy since there were no significant differences in ORR, OS, and PFS as compared with patients without FGFR2/3 mutations. However, the lack of response to subsequent Erdafitinib in all FGFR‐altered UCs suggests that attempting FGFRi prior to EV may be more reasonable, but larger, prospective studies are required to verify this. Moreover, the approval of sacituzumab govitecan further increases the complexity of sequencing agents for *FGFR2/3* altered patients. Therefore, the activity, optimal sequencing, and combinations of new agents and regimens in *FGFR2/3* that altered patients require further assessment.

## AUTHOR DISCLOSURES

Praful Ravi has no disclosures.

Toni K. Choueiri reports the following:

• Research (Institutional and personal): AstraZeneca, Alexion, Bayer, Bristol Myers‐Squibb/ER Squibb and sons LLC, Cerulean, Eisai, Foundation Medicine Inc., Exelixis, Ipsen, Tracon, Genentech, Roche, Roche Products Limited, F. Hoffmann‐La 2 Roche, GlaxoSmithKline, Lilly, Merck, Novartis, Peloton, Pfizer, Prometheus Labs, Corvus, Calithera, Analysis Group, Sanofi/Aventis, Takeda.

• Honoraria: AstraZeneca, Alexion, Sanofi/Aventis, Bayer, Bristol Myers‐Squibb/ER Squibb and sons LLC, Cerulean, Eisai, Foundation Medicine Inc., Exelixis, Genentech, Roche, Roche Products Limited, F. Hoffmann‐La Roche, GlaxoSmithKline, Merck, Novartis, Peloton, Pfizer, EMD Serono, Prometheus Labs, Corvus, Ipsen, Up‐to‐Date, NCCN, Analysis Group, NCCN, Michael J. Hennessy (MJH) Associates, Inc (Healthcare Communications Company with several brands such as OnClive, PeerView and PER), Research to Practice, L‐path, Kidney Cancer Journal, Clinical Care Options, Platform Q, Navinata Healthcare, Harborside Press, American Society of Medical Oncology, NEJM, Lancet Oncology, Heron Therapeutics, Lilly.

• Consulting or Advisory Role: AstraZeneca, Alexion, Sanofi/Aventis, Bayer, Bristol Myers‐Squibb/ER Squibb and sons LLC, Cerulean, Eisai, Foundation Medicine Inc., Exelixis, Genentech, Heron Therapeutics, Lilly, Roche, GlaxoSmithKline, Merck, Novartis, Peloton, Pfizer, EMD Serono, Prometheus Labs, Corvus, Ipsen, Up‐to‐Date, NCCN, Analysis Group, Pionyr, Tempest.

• No speaker's bureau.

• Stock ownership: Pionyr, Tempest.

• No leadership or employment in for‐profit companies. Other present or past leadership roles: Director of GU Oncology Division at Dana‐Farber and past President of medical Staff at Dana‐Farber), member of NCCN Kidney panel and the GU Steering Committee, past chairman of the Kidney Cancer Association Medical and Scientific Steering Committee).

• Patents, royalties or other intellectual properties: ‐International Patent Application No. PCT/US2018/12209, entitled “PBRM1 Biomarkers Predictive of Anti‐Immune Checkpoint Response,” filed January 3, 2018, claiming priority to U.S. Provisional Patent Application No. 62/445,094, filed January 11, 2017 ‐International Patent Application No. PCT/US2018/058430, entitled “Biomarkers of Clinical Response and Benefit to Immune Checkpoint Inhibitor Therapy,” filed October 31, 2018, claiming priority to U.S. Provisional Patent Application No. 62/581,175, filed November 3, 2017.

• Travel, accommodations, expenses, in relation to consulting, advisory roles, or honoraria.

• Medical writing and editorial assistance support may have been funded by Communications companies funded by pharmaceutical companies (ClinicalThinking, Envision Pharma Group, Fishawack Group of Companies, Health Interactions, Parexel, Oxford PharmaGenesis, and others).

• The institution (Dana‐Farber Cancer Institute) may have received additional independent funding of drug companies or/and royalties potentially involved in research around the subject matter.

• CV provided upon request for scope of clinical practice and research.

• Mentored several non‐US citizens on research projects with potential funding (in part) from non‐US sources/Foreign Components.

David Kwiatkowski receives research support from Genentech and Revolution Medicines; and is a consultant to Novartis, Genentech, and AADi.

Guru Sonpavde reports the following:

- **Advisory Board:** BMS, Genentech, EMD Serono, Merck, Sanofi, Seattle Genetics/Astellas, Astrazeneca, Exelixis, Janssen, Bicycle Therapeutics, Pfizer, Immunomedics/Gilead, Scholar Rock, G1 Therapeutics.
- **Research Support to Institution:** Sanofi, Astrazeneca, Immunomedics/Gilead, QED, Predicine, BMS.
- **Steering committee of studies**: BMS, Bavarian Nordic, Seattle Genetics, QED, G1 Therapeutics (all unpaid), and Astrazeneca, EMD Serono, Debiopharm (paid).
- **Data safety monitoring committee**: Mereo.
- **Travel costs**: BMS (2019), Astrazeneca (2018).
- **Writing/Editor fees**: Uptodate, Editor of Elsevier Practice Update Bladder Cancer Center of Excellence.
- **Speaking fees**: Physicians Education Resource (PER), Onclive, Research to Practice, Medscape (all educational).

## AUTHOR CONTRIBUTIONS

Elio Adib, Talal El‐Zarif, and Guru P. Sonpavde contributed to the conception and design of the study. William P. Skelton IV, Catherine Curran, Dory Freeman, Elie W. Akl, Amin Nassar, and Praful Ravi contributed to data collection. Elio Adib and Talal El‐Zarif conducted the data analysis. Elio Adib, Rohit K. Jain, Charlene Mantia, David J. Kwiatkowski, Toni K. Choueiri, and Guru P. Sonpavde supervised the work. Elio Adib, Talal El‐Zarif, and Guru P. Sonpavde wrote the manuscript draft. All authors reviewed and approved of the manuscript.

## Supporting information


**Table S1.** Baseline demographic and clinical characteristics of 60 mUC patients treated with EV with corresponding *FGFR2/3* status.

**Table S2.** Baseline demographic and clinical characteristics of 60 mUC patients treated with EV according to *FGFR2/3* status.

**Table S3.** Multivariable Cox regression for overall survival and progression‐free survival.
